# Effects of cashew gum and nanoparticles on cooled stallion semen

**DOI:** 10.1186/s13028-020-00530-6

**Published:** 2020-06-18

**Authors:** Kahynna Cavalcante Loureiro, Isabel Bezerra Lima-Verde, Anders Johannisson, Theodoros Ntallaris, Alessandro Jager, Petr Štěpánek, Marcelo da Costa Mendonça, Patrícia Severino, Jane M. Morrell

**Affiliations:** 1 Laboratory of Nanotechnology and Nanomedicine (LNMED), Institute of Technology and Research (ITP), Av. Murilo Dantas 300, Aracaju, 49010-390 Brazil; 2 Postgraduate Program in Industrial Biotechnology (PBI), Tiradentes University (UNIT), Av. Murilo Dantas 300, Aracaju, 49032-490 Brazil; 3 Department of Supramolecular Polymer Systems, Institute of Macromolecular Chemistry, Heyrovského námestí 2, 162 06 Prague 6, Czech Republic; 4 Department of Clinical Sciences, Swedish University of Agricultural Sciences, Box 7054, 75007 Uppsala, Sweden

**Keywords:** Cashew gum, Cooling, Nanoparticles, Semen, Stallion

## Abstract

**Background:**

Cryopreservation of stallion spermatozoa tends to cause plasma membrane damage due to the low ratio of cholesterol to phospholipids. Gums have been suggested as an alternative cryoprotectant to glycerol for stallion spermatozoa. Therefore, the present experiment was designed to verify whether the effect of addition of cashew gum (CG), or nanoparticles (NP) containing CG, to the extender before cooling on sperm quality in stallion semen. Ejaculates from 6 stallions were extended and split between six treatment groups (control, a-tocopherol [TOC], CG1, CG0.5, NP1 and NP0.5), stored in cryotubes at 4 °C.

**Results:**

Aliquots were analysed by computer-assisted sperm motility analysis on the day of collection, and after 24 h and 48 h of cold storage. After 48 h, the total motility with NP1 (78.53 + 6.31%) was similar to control 85.79 + 6.31% at 0 h. The same pattern was observed for progressive motility. Membrane integrity assessed by flow cytometer was similar between control, TOC and G1 at all storage times. The DNA fragmentation in the control group increased at all time points, whereas chromatin integrity was maintained after 24 h in TOC and NP0.5 compared to 0 h. There was no increase in the proportion of live spermatozoa producing hydrogen peroxide, but there was a tendency for an increased proportion of spermatozoa in the live superoxide category in CG1 after 24 h cooled storage.

**Conclusions:**

The addition of CG or CG-derived NP to extender for stallion semen was not harmful to the sperm cells.

## Background

Cooled stallion semen is widely used for equine artificial insemination since fertility rates are higher than frozen semen [1], even when the semen is used 36 h after collection [2]. The plasma membrane in stallion spermatozoa has a low ratio of cholesterol to phospholipids, resulting in susceptibility to cold shock during storage at low temperatures [3]; thus cooling the semen results in better sperm survival than cryopreservation. Thermal shock due to changes in temperature may cause injuries to the sperm membrane. Other functional and structural parameters such as motility, DNA integrity, production of reactive oxygen species (ROS) and changes in the acrosome [4] also occur, thus decreasing the survival and fertilizing ability of spermatozoa.

Different substances and compounds can be added to semen extenders to improve sperm quality. Gums are hydrocolloids that could be interesting for semen preservation; some studies have shown positive effects of different gums on cooled and cryopreserved semen. Ali et al. [5] showed the action of gum arabic on the preservation of cooled stallion semen, whereas Gastal [6] successfully demonstrated the cryoprotectant potential of xanthan gum on the membrane integrity of ovine spermatozoa. Furthermore, this gum did not affect important parameters such as membrane integrity and motility of human spermatozoa [7]. Other hydrocolloids, such as guar and agar, were used in the cryopreservation of human semen [7], improving sperm morphology and integrity of plasma and acrosomal membranes.

Cashew gum (CG) is extracted from the trunk of the cashew tree (*Anacardium occidentale* L.), widely present in northeastern Brazil. Some studies showed that CG exhibits anti-inflammatory [8], gastroprotective [9], antimicrobial [10, 11] and antidiarrheal [12] properties. It has been successfully used in the food and beverage industry as a colloidal stabilizer thickening as well as a gelling agent, in a similar fashion to other hydrocolloids, being a low cost compound, odorless, tasteless and nontoxic [13]. Due to these characteristics and the previous results obtained when different hydrocolloids were added to semen extenders, we hypothesized that CG can act as protectant during the cooling process of stallion semen.

An alternative additive could be to use nanoparticles (NP) produced from CG, due to their biocompatibility and low cytotoxicity. These NP bind to the cell membrane to deliver antioxidant substances, facilitating their diffusion through the sperm membrane. However, properties such as sperm morphology, size and superficial charge must be carefully evaluated to ensure quality, safety and efficacy, if these NP are to be used in semen extenders [14, 15]. Some studies have shown the successful use of NP to improve bovine [16], swine [17] and rooster [16] semen quality.

Thus, our aim in this study was to evaluate the effects of addition of CG, or NP containing CG, to the extender before cooling on sperm quality in stallion semen.

## Methods

### Study design

Semen was collected from six Warmblood stallions during the breeding season (July 2018) at a commercial stud (Lövsta Stud, Upplands Väsby, Sweden), using an artificial vagina according to standard husbandry practices. The stallions were aged between 3 and 18 years old (stallion 1–8 years old; stallion 2–14 years old; stallion 3–12 years old; stallion 4–43 years old; stallion 5–8 years old; stallion 6–18 years old), and were of known fertility. Immediately after collection, each semen sample was extended in Kenney’s extender (100 mL distilled water; 4.9 g glucose; 2.4 g skimmed milk) to a sperm concentration of 200 × 10^6^ spermatozoa/mL. The extended samples were transported to the laboratory at the Swedish University of Agricultural Sciences (Uppsala, Sweden) in an insulated box at 6 °C.

At the laboratory the sperm suspensions were further diluted 1:1 (final concentration 100 × 10^6^ spermatozoa/mL) in Kenney’s extender containing CG, NP or α-tocopherol (TOC) according to the experimental group, as follows: Control (Kenney’s extender); TOC (Kenney’s extender + α-tocopherol 2 mM); G1 (Kenney’s extender + cashew gum 1 mg/mL); G0.5 (Kenney’s extender + cashew gum 0.5 mg/mL); NP1 (Kenney’s extender + 1 mg/mL nanoparticles containing cashew gum and α-tocopherol); NP0.5 (Kenney’s extender + 0.5 mg/mL nanoparticles containing cashew gum and α-tocopherol). The samples were stored in cryotubes at 4 °C and analyzed on the day of collection and after 24 and 48 h.

The nanoparticles were prepared by nanoprecipitation with 1.0% of CG and 10% (w/w) of α-tocopherol, based on our previous study (Loureiro et al., unpublished data). Briefly, 1 mg/mL of CG was dissolved in an aqueous solution using magnetic stirring (Kasvi, K40-1820 H) for 30 min to obtain the aqueous phase. The organic phase was composed of 10% α-tocopherol dissolved in ethanol under magnetic stirring (Kasvi, K40-1820 H) for 30 min. After that, the organic phase was poured into the aqueous phase maintaining magnetic stirring for 30 min. The solvent was evaporated in a rotavapor at 35 °C, followed by the lyophilization process. The concentration of α-tocopherol in the experimental group TOC (10%) was chosen from previous studies [2].

### Sperm concentration

The sperm concentration in all samples was evaluated using a Nucleocounter-SP 100 cell counter (Chemometec, Allerød, Denmark) [18]. Briefly, an aliquot of 50 μL from each sample was mixed with 5 mL of reagent S100 in a sample cup. A cassette containing propidium iodide (PI) was filled with the mixture before inserting it into the Nucleocounter-SP100. The total cell count was displayed after 30 s.

### Computer-assisted sperm analysis (CASA)

CASA was performed using a SpermVision analyzer (Minitűb GmbH, Tiefenbach, Germany), connected to an Olympus BX 51 microscope (Olympus, Tokyo, Japan) with a heated stage (38 °C). The microscope was fitted with a 20× objective and a ×10 eyepiece; the camera operated at 60 frames per second. Aliquots of 5 μL from the sperm samples were pipetted on to a warm glass slide and a coverslip was placed on top. Sperm motility was analyzed in eight fields (at least 1000 spermatozoa) using the software program (SpermVision) with settings adjusted for stallion spermatozoa. Particles with an area ranging from 20 to 100 μm^2^ were included in the analysis. Sperm were considered be immotile if average path velocity (VAP) < 20, and locally motile if VAP > 20 and < 30, straightness (STR) < 0.5, curvilinear velocity (VCL) < 9 [19].

### Flow cytometry

Flow cytometry assays were performed according to a previous study, with minor modifications [19], using the staining protocols provided in the next sections. The flow cytometer used to evaluate the spermatozoa in all assays was a FACSVerse (BDBiosciences, San José, CA, USA).

### Sperm plasma membrane integrity (MI)

The MI was evaluated using SYBR-14 and propidium iodide (PI) (LIVE/DEAD^®^ Sperm Viability Kit L-7011; Invitrogen™ Molecular Probes™, Eugene, Oregon, USA), according to the procedure described previously [19]. The stock solution of SYBR-14 (1 mM) was diluted 1:50 in CellWash (BD Biosciences) to prepare a fresh SYBR-14 working solution (0.02 mM). A 300 µL aliquot of 2 × 10^6^ spermatozoa/mL in CellWash sperm suspension was labeled with 0.6 µL SYBR-14 working solution and 3 µL PI; 2.4 mM), then incubated at 37 °C for 10 min in the dark. A total of 30.000 events was collected and quantified as proportions of the population. Excitation was induced by a blue laser (488 nm). Green fluorescence was detected with a fluorescence channel FL1 band-pass filter (527/32 nm), whereas red fluorescence was measured using a FL3 band-pass filter (700/54 nm). After gating, the spermatozoa were classified according to the degree of integrity of the plasma membrane, as membrane intact (SYBR14+-PI−), and membrane damaged (SYBR14+-PI+) or (SYBR14–PI+). Only the proportion of membrane intact spermatozoa are reported in this study.

### Mitochondrial membrane potential (MMP)

Evaluation of MMP was done by staining the spermatozoa with the lipophilic cationic mitochondrial membrane potential probe 5,5′,6,6′-tetrachloro-1,1′,3,3′-tetraethylbenzimidazolyl-carbocyanine iodide (JC-1, Invitrogen™ Molecular Probes™, Eugene, OR, USA), according to previous studies [20] with minor alterations. Briefly, an aliquot of 400 µL sperm suspension at a final concentration of 2 × 10^6^ spermatozoa/mL in CellWash was labeled with 1.2 µL of 3 mM JC-1 and incubated at 37 °C for 30 min in the dark. Excitation of JC-1 in stained cells was obtained by a blue laser (488 nm). Emitted fluorescence was detected using both FL1 band-pass (527/32 nm) and FL2 (586/42 nm) filters. Compensation values were 80% for FL2-FL1 and 0.5% for FL1-FL2. After gating to identify spermatozoa, 30,000 cells were evaluated and classified as having high respiratory activity MMP-H (orange fluorescence) or low respiratory activity MMP-L (green fluorescence).

### Reactive oxygen species (ROS)

The staining procedure was described previously [21]. Two sets of samples were prepared for each sperm suspension: an aliquot of the sperm suspension (2 million spermatozoa in 300 µL CellWash) was stained with 9 µL of 40 µM Hoechst 33258 (HO; Sigma, Stockholm, Sweden), 9 µL of 40 µM hydroethidine (HE; Invitrogen™ Molecular Probes™, Eugene, Oregon, USA), and 9 µL of 2 mM 2′, 7′-dichlorodihydrofluorescein diacetate (DCFDA; Invitrogen™ Molecular Probes™, Eugene, OR, USA). The samples were incubated at 38 °C for 30 min in the dark, then analyzed for ROS content using the flow cytometer. Excitation was performed with a blue laser (488 nm) and a violet laser (405 nm). The green fluorescence was detected with a FL1 band-pass filter (527/32 nm), red fluorescence was measured using a FL3 band-pass filter (700/54 nm), and green fluorescence excited by the violet laser was detected with a FL5 band-pass filter (528/45 nm). A total of 30,000 sperm-specific events was evaluated after gating out debris, and the spermatozoa were classified as follows: viable, superoxide-negative; viable, superoxide-positive; non-viable, superoxide-positive; viable, H_2_O_2_-negative; viable, H_2_O_2_-positive; non-viable, H_2_O_2_-negative; and non-viable, H_2_O_2_-positive.

### Sperm chromatin structure assay (SCSA)

This assay was performed according to the previously described method [18, 22]. Briefly, equal volumes of sperm suspension and Tris-sodium chloride-EDTA buffer (TNE) (0.15 mol/L NaCl, 0.01 mol/L Tris–HCl, 1 mmol/L EDTA, pH 7.4) were mixed together, snap-frozen in liquid nitrogen (LN_2_) and stored at − 80 °C until subsequent evaluation by flow cytometry. At the time of analysis, the frozen sperm suspensions were thawed on crushed ice and diluted 1:10 with TNE buffer immediately before staining. Subsequently, the spermatozoa were subjected to partial DNA denaturation by mixing 100 µL of sperm suspension with 200 µL of acid-detergent solution 0.17% Triton X-100 (Sigma-Aldrich, St. Louis, MO, USA); (0.15 mol/L NaCl, and 0.08 mol/L HCl; pH 1.2). After 30 s, the spermatozoa were labeled with 600 µL staining solution of acridine orange (AO) (Sigma-Aldrich) (6 μg/mL in 0.1 mol/L citric acid, 0.2 mol/L Na_2_HPO_4_, 1 mmol/L EDTA, 0.15 mol/L NaCl; pH 6.0). Within 3–5 min, the stained samples were evaluated using the flow cytometer. For each sample, at least 10,000 events were analyzed at a speed of 200 cells/s. Forward scatter (FSC), Side scatter (SSC), FL1 (green fluorescence) and FL3 (red fluorescence) were measured after excitation with a blue laser (488 nm). The DNA Fragmentation Index (%DFI—ratio of the proportion of cells with denatured, single-stranded DNA to total cells acquired, both with stable, double-stranded DNA, and denatured single-stranded DNA) was calculated for each sample using FCS Express version 5 (DeNovo Software, Glendale, CA, USA).

### Statistical analysis

All statistical analyses were performed with SAS^®^ software version 9.3 (SAS Institute, Books 24x7 Inc. SAS/ETS 9.3 user’s guide, SAS documentation, Cary, N.C.: SAS Institute Inc., 2011), using the MIXED procedure for linear mixed models. A repeated effect of time (hours) within animals was tested. The model used included the fixed effect of treatment (6 classes; control, G0.5, G1, NP0.5, NP1, TOC), the fixed effect of time (3 classes; 0 h, 24 h, 48 h), and the interaction between treatment and time. Animal was set as a random effect.

Least square means (Lsmeans, ± standard error of the mean, sem) estimated by the models were compared using the Scheffe adjustment for multiple-post ANOVA comparisons. When appropriate, a complementary t-test based on differences between paired values was applied. A contrast option was used to investigate different individual hypothesis.

## Results

The data from the CASA and Flow cytometry assays have been deposited in the Mendeley Data via the Datasets partner repository [23], with the dataset identifier 10.17632/xm5s94x2cx.2.

**Fig. 1:**
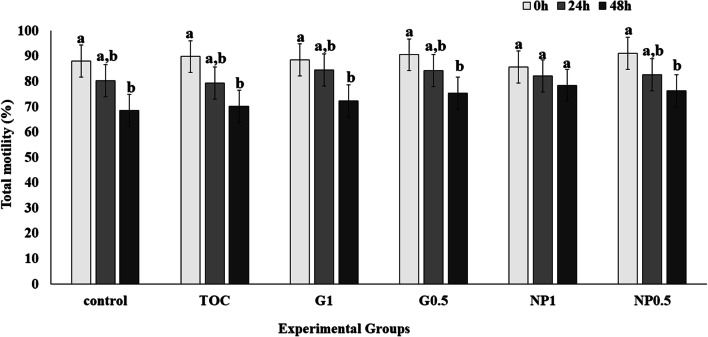
Effect of treatment on sperm total motility at 0 h, 24 h and 48 h. Control: Kenney’s extender; TOC: α-tocopherol; G1: cashew gum 1 mg/mL; G0.5: cashew gum 0.5 mg/mL; NP1: 1 mg/mL of nanoparticles; NP0.5: 0.5 mg/mL of nanoparticles; each bar represents the mean ± standard deviation of n = 6; a, b: Significant difference comparing the different storage times within the same treatment (P < 0.05)

There was a significant decrease in total motility (P < 0.05) after 48 h cooling compared with the day of collection (time 0 h), in the groups control, G1, G0.5 and NP0.5 (Fig. 1). However, when the semen samples were treated with NP 1 mg/mL, the total motility was preserved (P > 0.05) at all time points.

**Fig. 2:**
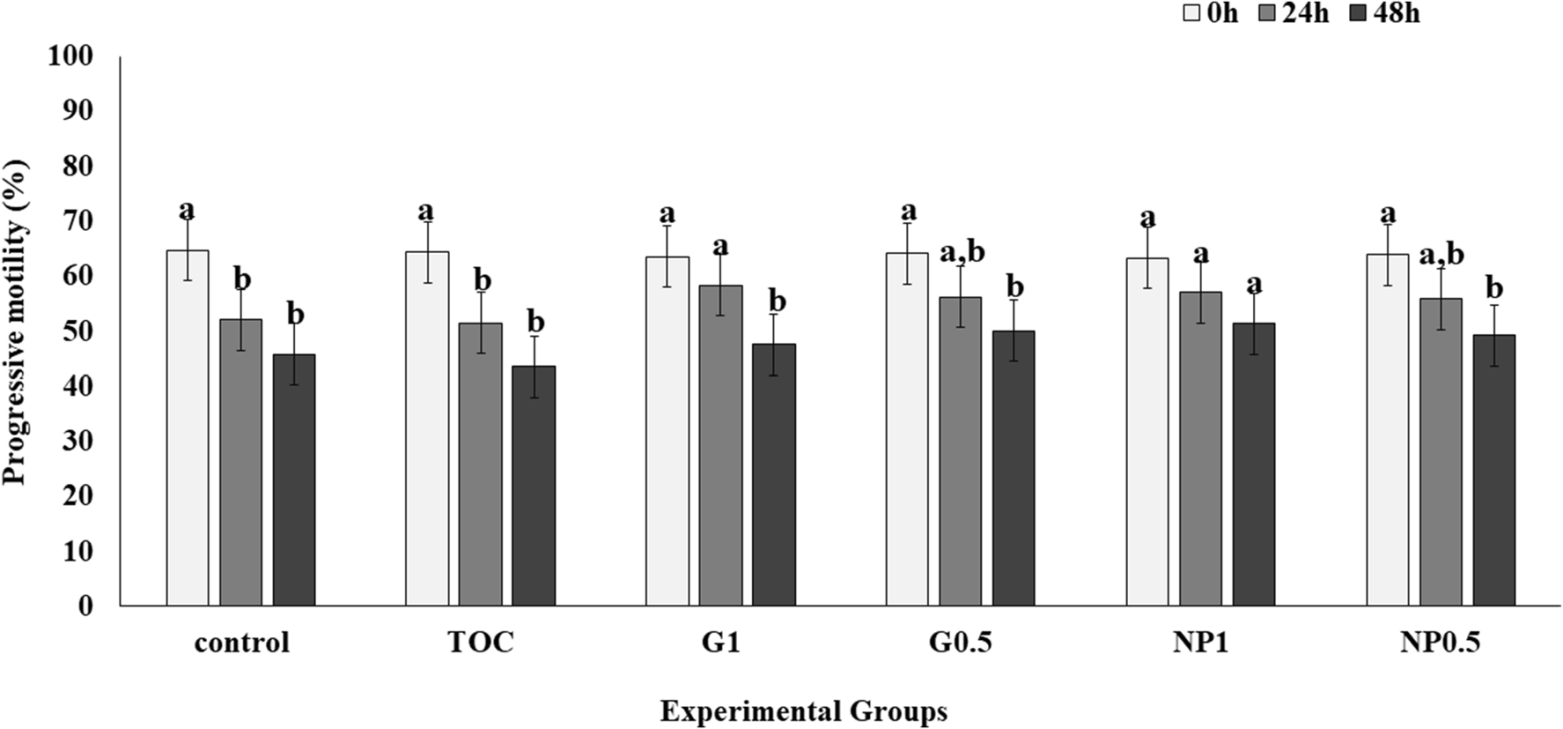
Effect of treatment on sperm progressive motility at 0 h, 24 h and 48 h. Control: Kenney’s extender; TOC: α-tocopherol; G1: cashew gum 1 mg/mL; G0.5: cashew gum 0.5 mg/mL; NP1: 1 mg/mL of nanoparticles; NP0.5: 0.5 mg/mL of nanoparticles; each bar represents the mean ± standard deviation of n = 6; a, b: Significant difference comparing different storage times within the same treatment (P < 0.05)

Control and TOC showed a decrease (P < 0.05) in progressive motility (Fig. 2) after 24 h of storage compared to 0 h, and G1 showed a decrease after 48 h compared to 24 h. As previously noted for total motility, the group NP1 showed similar progressive motility at all storage times (P > 0.05). Moreover, NP0.5 showed a decrease in progressive motility at 48 h of storage compared to 0 h (P < 0.05).

**Fig. 3:**
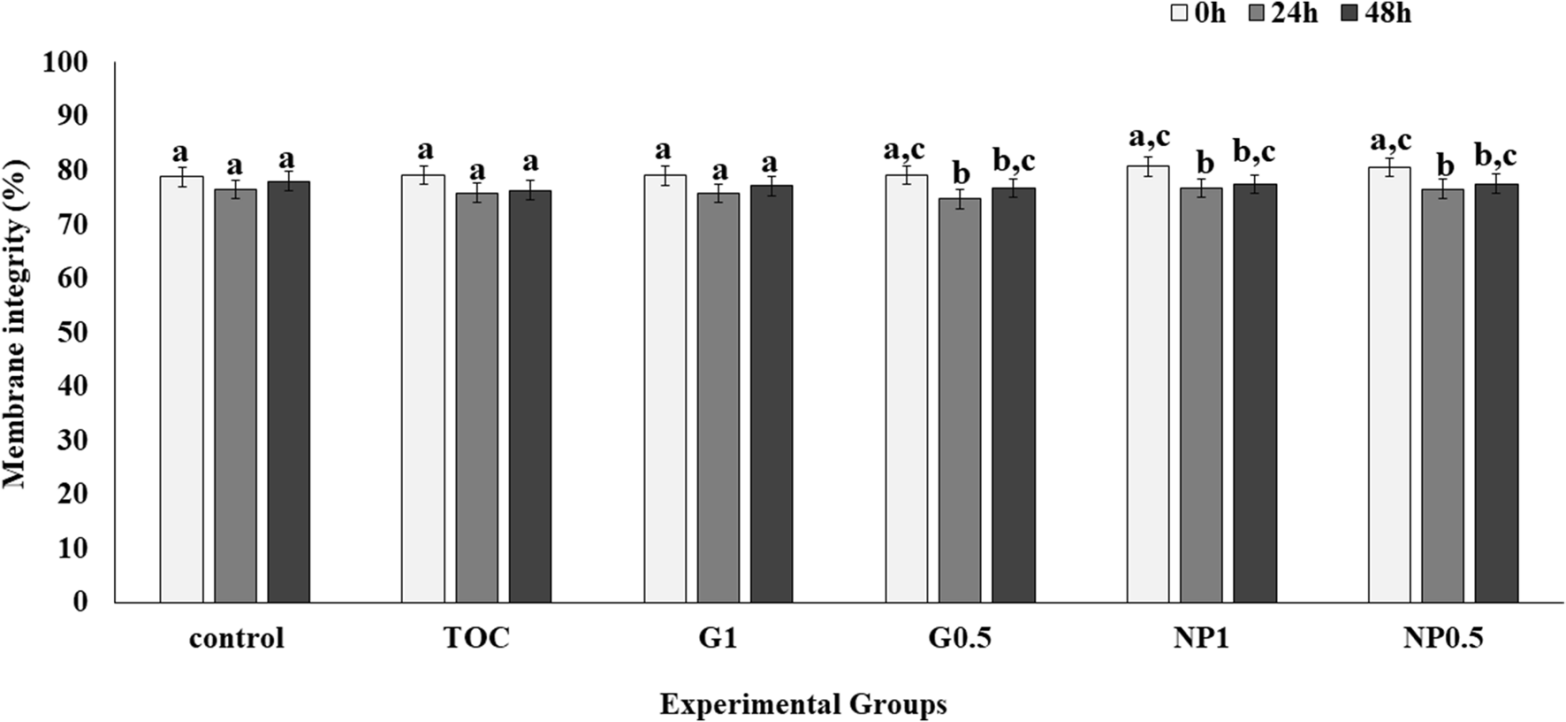
Evaluation of treatment on stallion sperm plasma membrane integrity at 0 h, 24 h and 48 h. Control: Kenney’s extender; TOC: α-tocopherol; G1: cashew gum 1 mg/mL; G0.5: cashew gum 0.5 mg/mL; NP1: 1 mg/mL of nanoparticles; NP0.5: 0.5 mg/mL of nanoparticles; each bar represents the mean ± standard deviation of n = 6; a–c Significant difference comparing different storage times within the same group (P < 0.05)

A significant decrease (P < 0.05) in MI was observed after 24 h compared to 0 h (Fig. 3) in groups G0.5, NP0.5 and NP1, although these levels then remained stable up to 48 h. However, MI was similar (P > 0.05) at all storage times in control, TOC and G1.

**Fig. 4:**
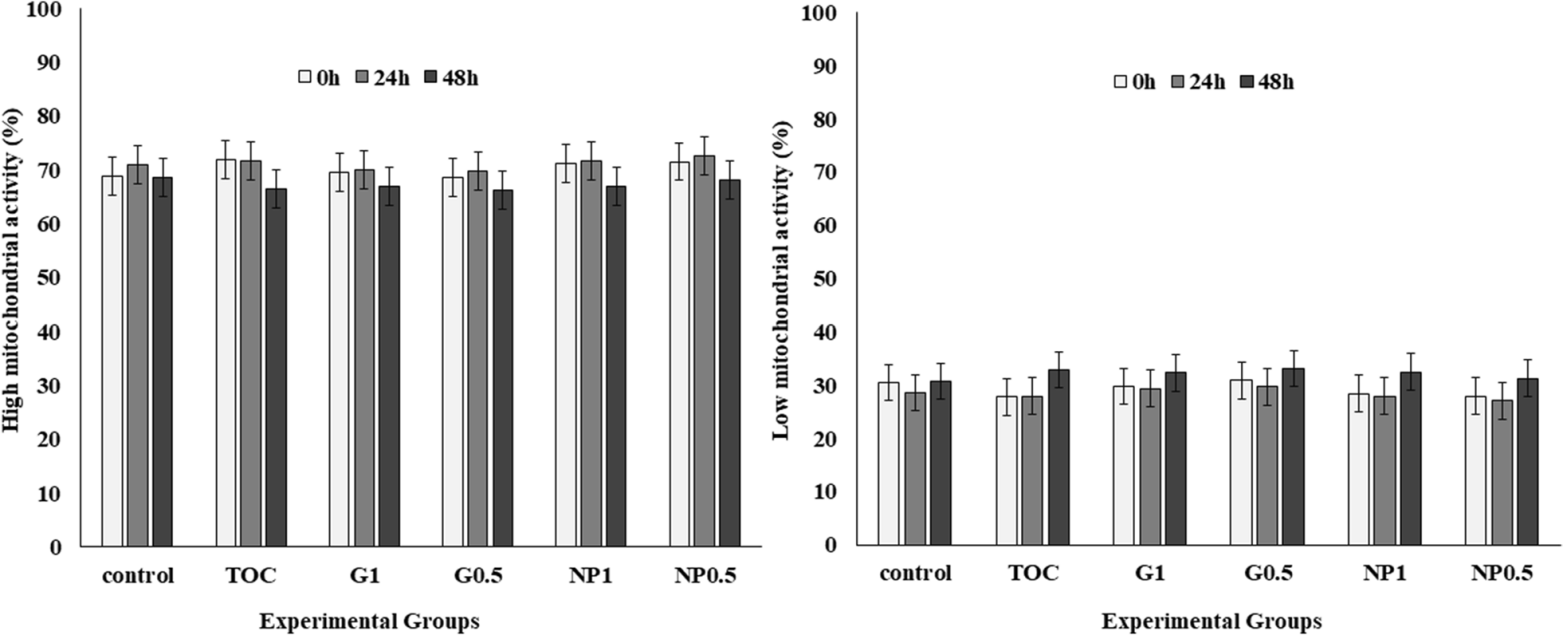
Effect of treatment on high mitochondrial activity in stallion sperm at 0 h, 24 h and 48 h. Control: Kenney’s extender; TOC: α-tocopherol; G1: cashew gum 1 mg/mL; G0.5: cashew gum 0.5 mg/mL; NP1: 1 mg/mL of nanoparticles; NP0.5: 0.5 mg/mL of nanoparticles; each bar represents the mean ± standard deviation of n = 6

MMP (Fig. 4) did not change among treatments or storage times (P > 0.05).

**Fig. 5:**
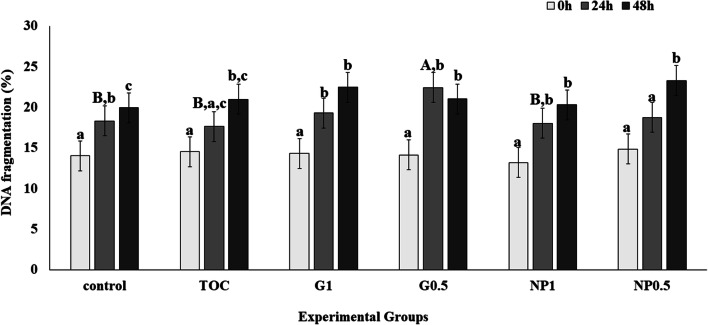
DNA fragmentation after treatment and storage for 0 h, 24 h and 48 h. Control: Kenney’s extender; TOC: α-tocopherol; G1: cashew gum 1 mg/mL; G0.5: cashew gum 0.5 mg/mL; NP1: 1 mg/mL of nanoparticles; NP0.5: 0.5 mg/mL of nanoparticles; each bar represents the mean ± standard deviation of n = 6; a–c: Significant difference comparing storage times within treatment (P < 0.05); A, B: Significant difference within the same storage time, comparing different treatments (P < 0.05)

The control group showed increased DNA fragmentation (P < 0.05) after 24 h and 48 h (Fig. 5). The DNA fragmentation was maintained (P > 0.05) after 24 h of cooled storage compared to 0 h in the groups TOC and NP0.5; a significant increase in %DFI (P < 0.05) in these same groups was observed only after 48 h. There was increased DNA fragmentation after 24 h cooled storage in the groups NP1, G0.5 and G1. After 24 h cooled storage, G0.5 showed higher %DFI than NP1, TOC and control groups (P < 0.05).

**Fig. 6:**
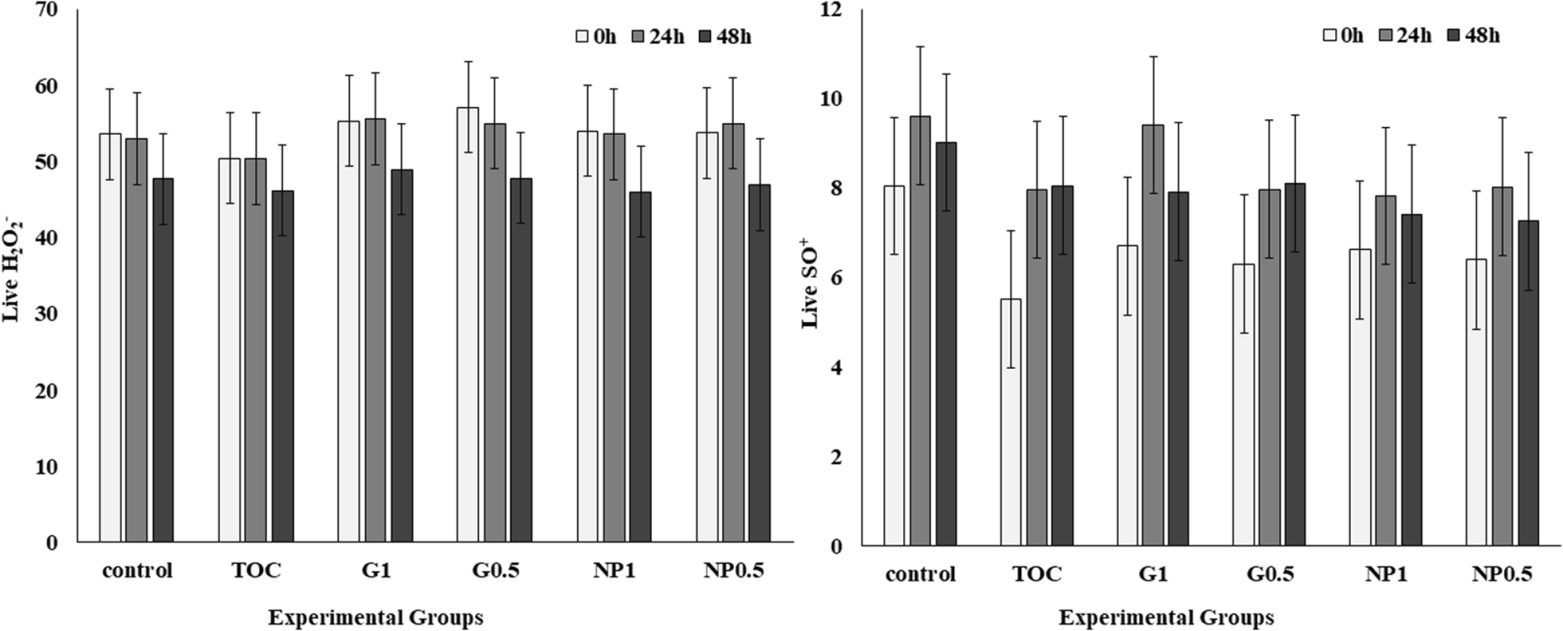
Flow cytometry analysis of reactive oxygen species (ROS) generation (H_2_O_2_ and SO^+^) of stallion spermatozoa, at 0 h, 24 h and 48 h in different treatments. Control: Kenney’s extender; TOC: α-Tocopherol; G1: Cashew gum 1 mg/mL; G0.5: Cashew gum 0.5 mg/mL; NP1: 1 mg/mL of nanoparticles; NP0.5: 0.5 mg/mL of nanoparticles; each bar represents the mean ± standard deviation of n = 6; a, b: Significant difference within the same group, comparing different storage times (P < 0.05)

There were no differences (P > 0.05) in live H_2_O_2_ in all treatments and at all evaluation time points (Fig. 6). However, there was a tendency (P = 0.06) to increased production of live SO^+^ in group G1 after 24 h, compared to 0 h.

## Discussion

The use of cooled semen samples is a common choice for commercial equine AI. However, this procedure still causes damage to the sperm cell during cold storage, and new alternatives are needed to improve the process. To our knowledge, this is the first study to use CG and NP derived from CG in order to improve the quality of cooled stallion semen.

In the present study, we observed that only the NP1 group was able to maintain both total and progressive motilities during the entire storage period (up to 48 h) (Figs. 1 and 2). In contrast to our findings, Pugliesi et al. [3] showed decreased sperm motility when using gum arabic in different concentrations in equine semen cooled for up to 96 h. According to these latter authors, this result is due to the increased viscosity that usually occurs when a hydrocolloid is added to an aqueous solution. In our study, no viscosity changes were observed in the tested media, which may have contributed to the different results in the two studies. In addition, the combination CG+NP+α-tocopherol in Kenney’s extender may have been ideal for maintaining motility throughout the cold storage period. Another factor to be considered is that in our experiment the seminal plasma was not removed from the collected samples, since the collection was performed according to standard husbandry practices in the commercial station. The benefits of removal of seminal plasma are debated since several studies reported different results in the presence or absence of seminal plasma during cooling or cryopreservation. It has also been reported that the use of extenders contributes significantly to reducing the negative effects of seminal plasma on sperm motility during cooled storage [19, 24]. Physiologically, it has been shown that sperm environment influences motion characteristics, and seminal plasma increases sperm motility and also promotes sperm activation [25].

Groups G0.5, NP0.5, NP1 showed a reduction in MI after 24 h, but then remained stable up to 48 h (Fig. 3). Previous studies also report a reduction in the values of some sperm parameters, such as MI, after cooling or cryopreservation and it seems that it is not possible to avoid some negative effects of cold shock [26]. However, in control, TOC and G1, MI was maintained up to 48 h of cooled storage. In the present study, we used Kenney’s extender, containing skimmed milk, which is important to prevent cold shock [27, 28]. The presence of casein micelles in the skimmed milk protects against the removal of phospholipids and cholesterol from the sperm plasma membrane during the cooling procedure [3].

Previous studies have shown that the addition of the antioxidant α-tocopherol to semen extender may improve cryopreservation or cooling of stallion semen [2, 29]; this antioxidant avoids the peroxidation of lipids in the plasma membrane, thus contributing to sperm preservation [30]. Gums provide protection against cold shock because of their physicochemical characteristics since they can retard or minimize the following processes: crystallization of water or sugar; gravitational sedimentation of suspended particles; loss of small molecules or ions due to the formation of an impermeable film; and syneresis in the gels, i.e. loss of water from the system [31–33]. In addition, these substances can bind to plasma membrane lipids, preventing efflux from the cells and contributing to the maintenance of cellular integrity [34].

Nanoparticles, due to their small size, may be easily integrated into the cells and migrate into different intracellular compartments. Moreover, NP show high stability, which enables them to be used over a long distance, thereby improving the delivery of some substances [35]. In addition, CG in controlled drug delivery systems, via NP, showed positive and successful results within different applications, such as antimicrobial activity [36, 37].

When mitochondrial activity was analyzed, all the treatments presented similar results at all storage times (Fig. 4). Such evaluation is a valuable test of sperm quality, since reduced mitochondrial activity is directly associated with sperm abnormalities [38, 39]. Our data suggest that GC and NP did not present toxic effects to sperm cells, considering that all the experimental groups were similar to the control, where only Kenney’s was used as extender.

The %DFI in the control group was increased when all the cooling periods were compared. The groups TOC and NP0.5 maintained DFI up to 24 h when compared to 0 h, but showed an increase at 48 h. However, NP1, G0.5 and G1 groups showed increased %DFI after 24 h but levels were then maintained up to 48 h (Fig. 5). The %DFI can be used to assess sperm DNA damage and can be directly associated with fertility [26, 40].

Some ROS such as H_2_O_2_ can increase sperm oxidative stress, which can cause cell damage, impairment of sperm fertility, and pathological conditions [26, 41, 42]. The storage of cooled stallion semen for long periods can induce sperm apoptosis due to oxidative stress and Johannisson et al. [21] showed that ROS can be a determinant factor of fertility reduction in stallions. However, our results showed similar live H_2_O_2_ levels in all treatments, suggesting that neither the extender, nor the gum, the nanoparticles or the tocopherol were harmful to spermatozoa by the production of this category of ROS (Fig. 6). It is already known that CG has a non-toxic heteropolysaccharide complex [11], which may have contributed to this result. Furthermore, there was a tendency for an increased proportion of spermatozoa in the live SO^+^ category in G1 after 24 h cooling, suggesting more metabolically active cells in this group [21].

## Conclusions

Our results suggest that CG and its NP can be used for cryopreservation of stallion semen and did not present harmful effects to sperm cells, at least in the concentrations used in this study. However, further investigations are necessary to verify the effects of other concentrations of CG and NP, sperm fertilizing ability, freezability and toxicity of the tested compounds.

## Data Availability

The original dataset generated during the current study are available in the Mendeley Data repository, 10.17632/xm5s94x2cx.2.

## Abbreviations

CASA: Computer-assisted sperm analysis
CG: Cashew gum
CG1: Kenney’s extender + cashew gum 1 mg/mL
CG0.5: Kenney’s extender + cashew gum 0.5 mg/mL
%DFI: DNA fragmentation index
MI: Membrane integrity
MMP: Mitochondrial membrane potential
NP: Nanoparticles
NP1: Kenney’s extender + 1 mg/mL nanoparticles containing cashew gum and α-tocopherol
NP0.5: Kenney’s extender + 0.5 mg/mL nanoparticles containing cashew gum and α-tocopherol
PI: Propidium iodide
STR: Straightness
TNE: Tris-sodium chloride-EDTA buffer
TOC: α-Tocopherol
VAP: Average path velocity
VCL: Curvilinear velocity
